# Precision Medicine Approaches to Overcome Resistance to Therapy in Head and Neck Cancers

**DOI:** 10.3389/fonc.2021.614332

**Published:** 2021-02-25

**Authors:** Sandra Ortiz-Cuaran, Jebrane Bouaoud, Andy Karabajakian, Jérôme Fayette, Pierre Saintigny

**Affiliations:** ^1^ Univ Lyon, Université Claude Bernard Lyon 1, INSERM 1052, CNRS 5286, Centre Léon Bérard, Centre de Recherche en Cancérologie de Lyon, Lyon, France; ^2^ Department of Medical Oncology, Centre Léon Bérard, Lyon, France; ^3^ Department of Maxillofacial Surgery and Stomatology, Pitié-Salpêtrière University Hospital, Pierre et Marie Curie University, Sorbonne University, Paris, France

**Keywords:** head and neck squamous-cell carcinoma, resistance, chemotherapy, cetuximab, immunotherapy, targeted therapy

## Abstract

Head and neck squamous cell carcinoma (HNSCC) is the sixth most incident cancer worldwide. More than half of HNSCC patients experience locoregional or distant relapse to treatment despite aggressive multimodal therapeutic approaches that include surgical resection, radiation therapy, and adjuvant chemotherapy. Before the arrival of immunotherapy, systemic chemotherapy was previously employed as the standard first-line protocol with an association of cisplatin or carboplatin plus 5-fluorouracil plus cetuximab (anti-EFGR antibody). Unfortunately, acquisition of therapy resistance is common in patients with HNSCC and often results in local and distant failure. Despite our better understanding of HNSCC biology, no other molecular-targeted agent has been approved for HNSCC. In this review, we outline the mechanisms of resistance to the therapeutic strategies currently used in HNSCC, discuss combination treatment strategies to overcome them, and summarize the therapeutic regimens that are presently being evaluated in early- and late-phase clinical trials.

## Introduction

Head and neck squamous cell carcinoma (HNSCC) is the sixth most incident cancer worldwide, responsible for more than 700,000 cases worldwide per year and around 350,000 deaths, making it a particularly fatal disease (1).

Squamous cell cancers of the oral cavity, the pharynx, and the larynx (the most frequent) are linked to smoking and alcohol consumption, and squamous cell carcinomas of the oropharynx are most commonly associated with human papilloma virus (HPV) infection, especially for young or nonsmoker patients. The incidence of the latter is rising, mostly among men (2). Cigarette- or alcohol-related and HPV-induced cancers are described by the 2017 World Health Organization (WHO) as two different clinical entities with different oncogenic pathways and prognostics (3). Other anatomical localizations of head and neck cancers include the sinus cavities and nasal fossae, which are rare and rather linked to professional and environmental exposures.

More than half of HNSCC patients experience locoregional or distant relapse despite aggressive multimodal therapeutic approaches that include surgical resection (often with neck dissection), radiation therapy (exclusive or postoperative), and adjuvant chemotherapy given as a radiosensitizer (4). After relapse, treatment options are often limited due to a high risk of complications (e.g., fistulas, dysphagia, spinal cord myelopathy) if surgery or reirradiation are attempted. If a salvage surgery (with R0 resection) or reirradiation is indeed deemed unfeasible, then systemic treatment options (detailed in this review) are proposed. Before the arrival of immunotherapy, systemic chemotherapy was employed as the standard first-line protocol with an association of cisplatin or carboplatin and 5-fluorouracil plus cetuximab (anti-EFGR antibody), known as the EXTREME protocol, which confers a dismal median overall survival (OS) of around 10 months (5).

In this review, we outline the mechanisms of resistance to the therapeutic strategies currently used in HNSCC, discuss combination treatment strategies to overcome them, and summarize the therapeutic regimens that are presently being evaluated in early- and late-phase clinical trials.

## Mechanisms of Resistance to Chemotherapy

Chemotherapy is currently used as the therapeutic option for advanced HNSCC tumors (T3 or T4), concurrent to radiation, if surgical resection is deferred in the primary setting. For recurrent or metastatic disease and for cases in which first-line treatment with immunotherapy is not feasible, first-line systemic chemotherapy is advised with a protocol that includes cisplatin or carboplatin plus 5-fluorouracil and cetuximab (5, 6). Unfortunately, acquisition of chemotherapy resistance is common in patients with HNSCC and often results in local and distant failure.

### Cancer Stem Cells and EMT

Epithelial-to-mesenchymal transition (EMT) is a reversible embryonic transdifferentiation program that allows partial or complete transition from an epithelial to a mesenchymal state (7). Although EMT was initially considered to be involved in invasion and metastatic spread, its key role in the initiation and development of primary tumors as well as in resistance to therapy is also demonstrated (8).

Nasopharyngeal carcinoma (NPC) is a highly invasive head–neck cancer derived from the nasopharyngeal epithelium. Preclinical studies in NPC cells demonstrate that resistance to radiotherapy and adjuvant cisplatin (DDP) chemotherapy is associated with morphological and molecular marker changes consistent with EMT. Mechanistically, depletion of NEDD4 in resistant cells leads to a partial reversion of the EMT phenotype, suggesting that NEDD4 promotes EMT features and chemoresistance of NPC *in vitro* (9). In a subsequent study, analysis of parental HNE1 and cisplatin-resistant HNE1/DDP NPC cells reveals that the upregulation of miR-139-5p expression inhibits proliferation, invasion, migration, and EMT. In these cells, miR-139-5p expression levels positively correlate with DDP-induced apoptosis, suggesting that miR-139-5p is associated with DDP resistance in human NPC by modulating the EMT (10).

More recently, it was demonstrated that epithelial mesenchymal crosstalk (EMC), which constitutes the interaction of the tumor stroma and associated fibroblasts with epithelial cancer cells, induces a hybrid epithelial–mesenchymal phenotype in HNSCC cells that is associated with chemotherapy resistance, *via* IL-6/STAT3 pathway activation (11). Interestingly, transcriptome analyses of HNSCC cell lines reveals that STAT1 and STAT3 activation enable aldo-keto reductase family 1 member C1 (AKR1C1)-induced resistance to cisplatin, which can be overcome by ruxolitinib treatment (12).

Cisplatin-resistant oral squamous cell carcinoma (OSCC) cells exhibit an enriched putative cancer stem–like signature with increased expression of CD44 and Oct-4 and enhanced sphere-forming ability, coupled with the acquisition of an EMT phenotype. This study also reveals that, irrespective of drug treatment, cell migration is significantly increased in cisplatin-resistant cell lines compared with drug-sensitive cells. In line with these observations, bioinformatic analysis of miRNA–mRNA networks in cisplatin-resistant OSCC cells reveals the upregulation of ATP-binding cassette (ABC) transporter genes, genes associated with inhibition of apoptosis (e.g., BIRC family) and cancer stem cell (CSC) marker CD44 (13).

A subpopulation of CSCs characterized by high levels of CD44v3 and aldehyde dehydrogenase-1 (ALDH1) expression has been identified in HNSCC-derived HSC-3 cells and HNSCC patient samples. In HSC-3 cells, it is shown that hyaluronan (HA) stimulates the interaction of CD44v3 with Oct-4-Sox2-Nanog, which results in the nuclear translocation of these three CSC transcription factors. Notably, it is demonstrated that Oct-4-Sox2-Nanog– dependent activation of miR-302 promotes the upregulation of the survival proteins cIAP-1, cIAP-2, and XIAP, leading to self-renewal and cisplatin resistance. In this context, transfection with an anti-miR-302 inhibitor is shown to downregulate the expression of these survival proteins and to abrogate the HA-CD44v3–mediated sphere formation and chemoresistance (14). It is noteworthy that the histone methyltransferase DOT1L is also upregulated by HA in CSCs isolated from HSC-3 cells and results in the overexpression of RhoGTPases and survival proteins involved in cell invasion and cisplatin resistance (15).

Inhibition of the aldehyde dehydrogenase 1 family member A1 (ALDH1A1) in cisplatin-resistant HNSCC cells results in downregulation of CSC markers that are diminished in migratory, self-renewal, and tumorigenic potential and resensitizes HNSCC cells to cisplatin. These observations are further validated in four ex vivo explants from HNSCC patients in which combined treatment of cisplatin and NCT-501, a theophylline-based inhibitor of ALDH1A1, results in a significant decrease in proliferating cells as compared with monotherapy (16). In a subsequent study, gene set enrichment analysis identified enhanced FGF2 expression in cisplatin-resistant ALDHhigh/CD44high HNSCC cells. Pharmacological inhibition of FGF signaling using BGJ398 preferentially targeted the ALDHhigh/CD44high subpopulation, suggesting that FGFR signaling plays a key role in *in vitro* stemness and in cisplatin resistance in HNSCC cells (17).

Of note, preclinical studies show that long noncoding RNA FOXD2-AS1 regulates therapeutic resistance in laryngeal squamous cell carcinoma (LSCC) by acting as an upstream activator of STAT3, which is essential to maintain cancer stemness. In LSCC patients, FOXD2-AS1 expression was predictive of poor prognosis in chemotherapy-resistant patients (18).

Overall, these studies show that the acquisition of CSC properties and the transition to a mesenchymal phenotype mediate chemotherapy resistance of HNSCC.

### DNA Damage

Cisplatin triggers the formation of phosphorylated histone H2AX (γ H2AX)-positive foci at the site of DNA damage (19), dependent on ATR and *via* the activation of downstream CHEK1/2 (20). In HNSCC, as well as in other cancer types, altered DNA damage response signaling has been associated with resistance to chemotherapies (21).

Indeed, functional depletion of DDR effectors WDHD1, DSCC1, CSNK2B, POLR2I, and RAD54L in HNSCC cells treated with cisplatin results in decreased ATR serine/threonine kinase (ATR) phosphorylation and reduces cisplatin-induction of γ H2AX foci, suggesting that impaired DDR signaling is a driving mechanism of cisplatin resistance in HNSCC *in vitro* (21). Moreover, gene expression analysis of pretreatment biopsy specimens from 64 HNSCC patients treated with 5−FU/cisplatin identified that ERCC1 expression is a significant predictor of response to chemotherapy, further indicating that DNA repair is a pivotal mechanism implicated in response to chemotherapy in HNSCC (22).

At present, clinical trials are evaluating the efficacy of targeting DNA damage response in HNSCC. ATR acts as a DNA damage sensor, activating cell cycle checkpoint signaling upon DNA stress. Pharmacological inhibition of ATR using M6620 is currently being tested in combination with cisplatin and radiation therapy in the setting of locally advanced HNSCC (NCT02567422). Similarly, a modular, phase-I/Ib, open-label trial is ongoing to evaluate the efficacy of ceralasertib (AZD6738, ATR inhibitor) in combination with carboplatin (NCT02264678).

Along the same lines, because PARP is involved in DNA repair, inhibition of PARP may enhance the damaging effects of chemotherapy on tumor DNA. A phase-I/II study recently reported the safety and efficacy of veliparib, a PARP inhibitor, in combination with carboplatin-paclitaxel chemotherapy in patients with locoregionally advanced HNSCC (23). The WEE1 tyrosine kinase maintains genomic stability and regulates G2–M transition, particularly in p53-deficient tumors, protecting cells against replication stress and subsequent cell death. A phase-I clinical trial evaluating the triplet combination of AZD1775 (WEE1 inhibitor), cisplatin and docetaxel reported satisfactory results in terms of safety and tolerability as well as promising antitumor efficacy in patients with stage-III/IVB HNSCC (i.e., partial response in 5 patients and stable disease in 4 patients) (24).

### Epigenetic Modifications

Resistance to cisplatin-based chemotherapy can be also modulated by epigenetic alterations. Indeed, hypermethylation of the promoter CpG islands of the neurofilament light polypeptide (*NEFL*) gene is associated with resistance to cisplatin-based chemotherapy in HNSCC cell lines. Functional analyses showed that NEFL interacts with tuberous sclerosis complex 1 (TSC1) at the protein level. Because TSC1 is a negative regulator of the mTOR pathway, it is suggested that NEFL downregulation results in functional activation of the mTOR pathway and, thus, cisplatin resistance. Interestingly, in this study, analysis of more than 50 HNSCC patient samples evidenced that *NEFL* promoter hypermethylation predicted diminished OS and disease-free survival in patients treated with cisplatin-based chemotherapy (25). A recent clinical trial evaluated the impact of mTOR pathway inhibition in HNSCC patients in the neoadjuvant setting. This study reports that rapamycin treatment was well tolerated, reduced mTOR signaling (i.e., phosphorylation of S6, AKT, and 4EBP) and tumor growth, and resulted in significant clinical responses in 4/16 of patients (1 complete response, 3 partial responses, and 12 stable disease) (26).

Histone modifiers are essential for chromatin dynamics and gene expression, and their dysregulated function may alter gene regulation in favor of oncogenic growth. Elevated expression of p21-activated kinase 2 (PAK2), a binding partner of the Rho GTPases that are implicated in chromatin remodeling, cell proliferation, and apoptosis, is correlated with chemoresistance and is associated with the poor clinical outcome of HNSCC patients. Mechanistically, PAK2 upregulates c-Myc expression, which, in turn, transcriptionally activates and induces pyruvate kinase M2 (PKM2) expression, resulting in reduced aerobic glycolysis, proliferation, and chemotherapeutic resistance of HNSCC cells (27).

Small noncoding RNAs are also key effectors of transcriptional gene silencing in HNSCC. Analysis of global miRNA expression in CD44-expressing HNSCC cells reveals that miR-629-3p expression promotes cell migration and inhibits apoptotic cell death upon cisplatin treatment. Of note, miR-629-3p-transfected cells display a significant enrichment of gene sets associated with drug metabolism and EMT. Interestingly, the role of miR-629-3p in conferring resistance to cisplatin was also observed in a xenograft model, and the expression of miR-629-3p was associated with decreased survival in HNSCC patients, potentially suggesting a physio-pathological role of miR-629-3p in resistance to cisplatin in HNSCC (28).

Enhanced expression of miR-96-5p is shown to promote cell migration but not cell proliferation, in p53-mutant HNSCC cell lines and to drive resistance to radiotherapy and cisplatin-based chemotherapy *in vitro* (29). Of note, this study identified PTEN, a negative regulator of the intracellular levels of phosphatidylinositol-3,4,5-trisphosphate as a direct target of miR-96-5p through the binding to its cognate site on the 3’UTR of PTEN. Interestingly, functional experiments performed *in vitro* shows that PTEN depletion recapitulates the biological effects of miR-96-5p overexpression in HNSCC cells as they were less prone to cisplatin-induced cell killing (29).

At present, a noninterventional clinical trial (NCT03953443) is evaluating the impact of expression and epigenetic silencing of microRNAs for predicting therapeutic response and prognosis of HPV-negative HNSCC.

Further knowledge on the epigenetic alterations that promote HNSCC chemoresistance can open the possibility for the development of therapeutic strategies that can be used as an adjuvant therapy associated with conventional chemotherapeutic drugs to enhance treatment effectiveness.

### Evasion of apoptosis

The adaptive response to chemotherapy in HNSCC is modulated by changes in the expression of pro- or anti-apoptotic proteins and include defects in cellular responses caused by mutations of tumor suppressor gene *TP53* (30, 31).

Survivin (BIRC5), a member of the inhibitor of apoptosis (IAP) gene family, is shown to be significantly upregulated in HNSCC primary tumors and cell lines and to be particularly highly expressed in HPV-negative patients who generally respond poorly to cisplatin treatment (32). Immunohistochemical and mutational analyses on HNSCC biopsies from patients displaying high levels of nuclear survivin (BIRC5) identified the presence of the somatic mutation c.278T>C (p.Phe93Ser). Functional characterization of this mutant by ectopic expression and microinjection experiments revealed that it attenuates the cytoprotective activity of survivin against chemoradiation-induced apoptosis. Therefore, genetic inactivation of survivin may promote an increased therapy response in cancer patients (33).

Interestingly, pharmacological inhibition of survivin using the small molecule YM155, either as a single agent or in combination with cisplatin, evidenced a significant dose-dependent decrease in cell proliferation and the reversion of cisplatin resistance in *in vitro* and *in vivo* models of HNSCC. Mechanistically, YM155 induced a rapid reduction of survivin in the cytoplasm, which is key for its antiapoptotic function (32). Thus, survivin inhibition might potentially be a novel strategy to enhance the effectiveness of chemotherapy in HNSCC.

The x-linked inhibitor of apoptosis (XIAP) is an E3 ubiquitin protein ligase that functions through binding to tumor necrosis factor receptor–associated factors TRAF1 and TRAF2 to inhibit apoptosis. Analysis of XIAP expression in tumor samples from 60 patients with advanced HNSCC, before and after chemotherapy, evidenced that XIAP is a predictor of cisplatin response and prognosis for patients with advanced HNSCC. Interestingly, preclinical experiments show that inhibiting XIAP expression with siRNA in XIAP-high HNSCC cells markedly increased their sensitivity to cisplatin treatment (30). Of note, the dual cIAP/XIAP antagonist ASTX660 significantly delays growth of both HPV- and HPV+ human tumor xenografts in combination with radiotherapy.

Resistance to the activity of TNF-related apoptosis inducing ligand (TRAIL), an effector of tumor cell–specific apoptosis, is associated with HPV positivity in HNSCC *in vitro*. HPV-positive HNSCC cell lines were sensitized to TRAIL-induced cell death by bortezomib-mediated proteasome inhibition *via* the activation of caspases 8, 9, and 3; increased membrane expression of TRAIL-R2; and G2/M arrest. Of note, XIAP depletion also augmented HPV-positive HNSCC cell death in response to TRAIL alone and in combination with bortezomib (34).

### Tumor Microenvironment (TME)

HNSCC tumors are commonly associated with hypoxia, which is characterized by an acute or chronic decline in oxygen tension.

Activin receptor–like kinase (ALK)-1 represents a promising target for antiangiogenic therapy in solid tumors. activin receptor–like kinase-1 ligand trap (ALK1-Fc) is a chimeric protein consisting of the ALK1 extracellular domain fused to the Fc-part of an antibody. ALK1-Fc prevents the binding of BMP9 and BMP10 to the endothelial ALK1 receptor, which results in decreasing angiogenic responses (35). Therapeutic combination of ALK1-Fc with cisplatin is shown to inhibit tumor growth in HNSCC *in vivo* models more efficiently than chemotherapy alone. Treatment of mice with ALK1-Fc strongly decreased the microvascular density of tumors, increased the pericyte coverage of the remaining tumor vessels, and decreased the hypoxia within the tumor (36). Interestingly, results of an early-phase clinical trial show that the ALK1-Fc displayed promising antitumor activity in HNSCC patients with advanced refractory cancer (35).

Signaling *via* the SDF-1/CXCR4 axis, a chemokine-receptor pathway, is involved in cancer progression due to its roles in modulation of dendritic cells, enhanced matrix metalloproteinase activity, and the induction of TNF-alpha production and angiogenesis. Analysis of the expression of SDF-1 and CXCR4 in a cohort of 221 patients with locally advanced HNSCC evidenced that SDF-1 is associated with resistance to adjuvant radiotherapy concurrent with cisplatin-based chemotherapy (37). In this study, neither SDF-1 nor CXCR4 expression were associated with distant metastasis or with OS. The functional basis of these observations as well as the potential role of SDF-1/CXCR4 as a therapeutic target to overcome treatment resistance in HNSCC remains to be determined.

In preclinical models of OSCC, combination therapy of cisplatin and inhibitors of VEGFR (i.e., pazopanib and nintedanib) was more potent than treatment with chemotherapy alone (38). The efficacy and toxicity of docetaxel with or without vandetanib, an inhibitor of VEGFR, RET, and EGFR, was investigated in patients with advanced recurrent or metastatic HNSCC. This trial shows only a minor trend toward improved PFS for the combination arm (39). Of note, a current clinical trial is ongoing to evaluate the combination of atezolizumab (humanized IgG1 antibody against PD-L1) and bevacizumab (monoclonal antibody developed against VEGF) in patients with recurrent or metastatic HNSCC (ATHENA, NCT03818061).

The TME also constitutes a reservoir of cancer-associated fibroblasts (CAFs) which, in a close crosstalk with tumor cells, enhance the production of growth factors, cytokines, chemokines, and inflammatory mediators to promote tumor growth (40). CAFs are observed in both primary and metastatic HNSCC, and oral CAFs are reported to acquire rapid growth and increased proliferation and viability compared with normal oral fibroblasts (40). CAF-secreted paracrine factors increase HNSCC migration, invasion, and proliferation *in vitro* and promote tumor growth and metastases *in vivo* (i.e., orthotopic floor-of-the-mouth tumor model) (41).

CAFs are also known to mediate resistance to anticancer drugs in HNSCC. In HNSCC cell lines, culture with conditioned medium from a tumor cell/CAF coculture induced cisplatin resistance and increased their colony-formation capacity (11). Interestingly, exosomal miR-196a released by CAFs targets CDKN1B and ING5 and, thus, confers cisplatin resistance *in vitro* (42). Interestingly, in this context, high levels of plasma exosomal miR-196a are clinically correlated with poor OS and chemoresistance in HNSCC patients. In line with these observations, it is demonstrated that, in OSCC patients, CAFs secrete increased levels of midkine (a heparin-binding growth factor that promotes carcinogenesis and chemoresistance) and abrogated cisplatin-induced cell death (43). Finally, analysis of tumor specimens obtained from 60 OSCC patients who underwent surgery following 5-fluorouracil-based chemoradiotherapy revealed that higher numbers of CAFs and tumor-associated macrophages (TAMs) were significantly correlated with a poor prognosis, suggesting their potential as biomarkers for predicting the clinical response to 5-FU-based chemoradiotherapy (44).

Understanding how CAFs contribute to drug resistance, proliferation, invasion, and metastasis might open up new strategies for the diagnosis, prognosis, and therapy of HNSCC.

## Mechanisms of Resistance to Cetuximab

Initially described in 1962 by Cohen (45, 46), the epidermal growth factor receptor (EGFR) is a transmembrane receptor with tyrosine kinase activity (47). Several ligands bind specifically EGFR (e.g., epidermal growth factor [EGF], tumor growth factor-alpha [TGF-alpha], and amphiregulin), and others (betacellulin, heparin-binding growth factor [HB-EGF], and epiregulin) bind to both EGFR and ErbB4 (48–50). Ligand binding induces the homo- or hetero-dimerization of EGFR, which is followed by the activation of downstream signaling, mainly *via* the RAS–RAF–MEK–ERK, the PI3K–AKT–mTOR, and the JAK–STAT cascades (51). These pathways are involved in the carcinogenesis and invasiveness of many cancer types (52).

Because EGFR is overexpressed in 80%–90% of HNSCC cases, tumors are often addicted to EGFR signaling for sustained survival and proliferation, and this overexpression is correlated with poor prognosis and treatment outcomes (53–55), therapies targeting EGFR have been widely evaluated for HNSCC (56–58): first, intravenous anti-EGFR antibodies that bind to the extracellular domain of the receptor causing its internalization to prevent its activation by other ligand–receptor interactions (59) while favoring antibody-dependent cell-mediated cytotoxicity (i.e., ADCC, which refers to the linking to innate and adaptive antitumor immune responses *via* NK cells and antigen-presenting cells that lead to EGFR-specific T cells) (60–63) and ii-oral EGFR tyrosine kinase inhibitors (TKI) binding to the intracellular domain of EGFR inhibiting its autophosphorylation (blocking of the ATP binding to the intracellular tyrosine kinase domain of EGFR) and downstream signaling (56, 64, 65).

Cetuximab (CTX), a monoclonal antibody targeting the EGFR extracellular domain, is to date the only targeted therapy that has demonstrated benefits in OS in combination with both radiotherapy for patients with locally advanced HNSCC (66) and chemotherapy (platinum, 5-FU, and CTX) as the first-line treatment of patients with recurrent and/or metastatic HNSCC (5, 67). Of note, CTX has never proven to be effective postoperatively (56, 58, 68).

**Figure 1:**
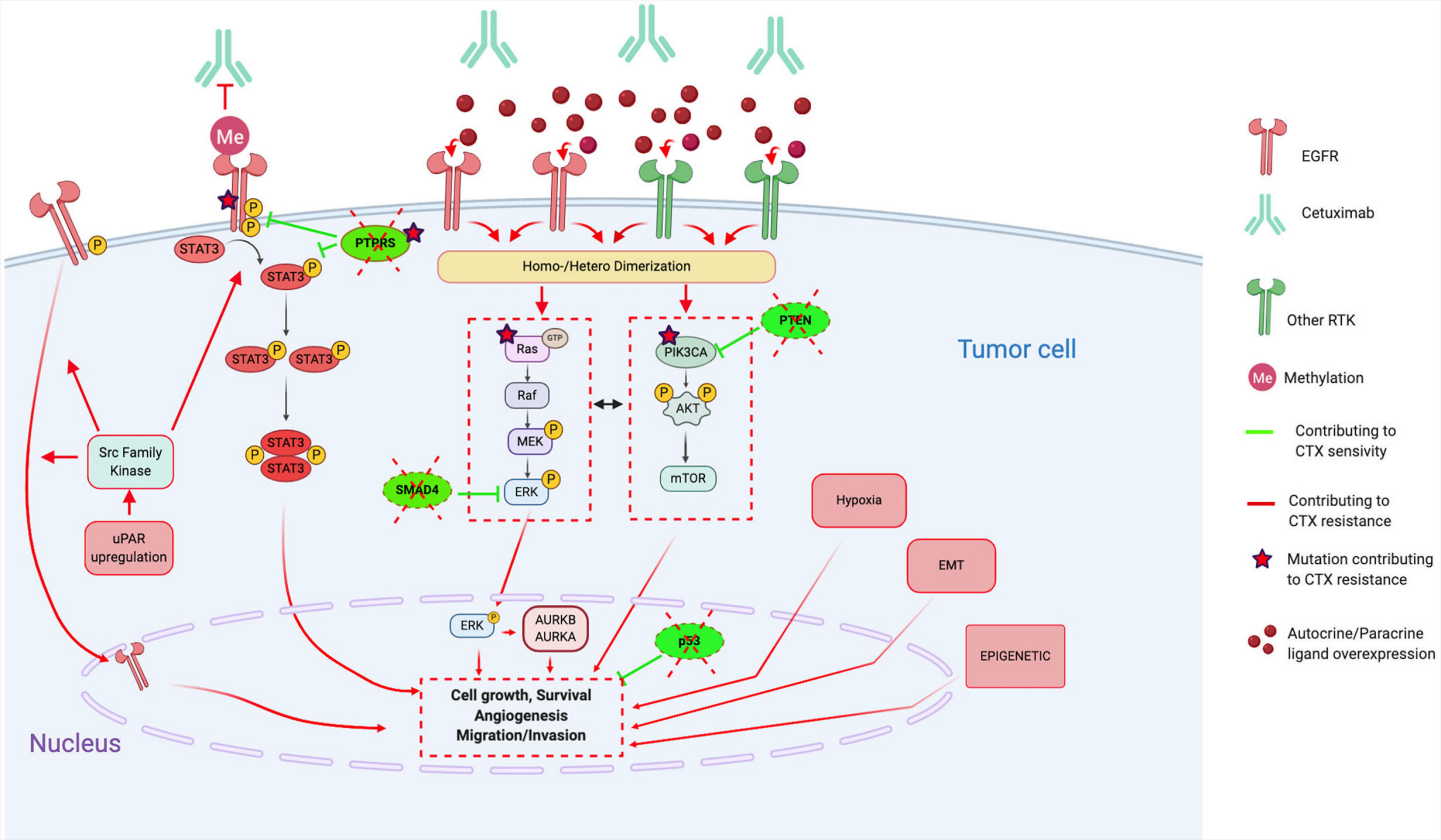
Molecular mechanisms contributing to Cetuximab resistance, in particular through alterations of the EGFR pathways, activation of bypass pathways and alterations of downstream signaling effectors. Red lines and arrows show mechanisms contributing to Cetuximab resistance, and green lines and arrows show mechanisms contributing to Cetuximab sensitivity. (CTX, Cetuximab; EGFR, Epidermal Growth Factor Receptor; RTK, Tyrosine Kinase Receptor; EMT, epithelial-mesenchymal-transition; uPAR, urokinase-type plasminogen activator receptor; STAT3, signal transducer and activator of transcription 3; PTPRS, Transmembrane Protein Tyrosine Phosphatase RPTPsigma; PTEN, phosphatase and tensin homolog; AURKA, Aurora Kinase A; AURKB, Aurora Kinase B).

Despite our better understanding of HNSCC biology (51, 69–71), no other molecular-targeted agent has been approved for HNSCC (12). Furthermore, CTX has shown limited efficacy in HNSCC with an overall response rate of 10%–20%, contrasting with the high rates of EGFR overexpression (51, 72). This underlines the existence of resistance mechanisms, remaining unresolved, but for which several hypotheses have been proposed (48, 56, 64, 67, 73–81) (4, 12, 21, 24, 29–37). The different type of resistance mechanisms to CTX could be defined as follows: alterations of the EGFR-ligand binding, alterations of the EGFR downstream signaling effectors, parallel/bypass pathway activation, alterations of proteins involved in classic cancer pathways, EMT, epigenetic alterations and establishment of an immunosuppressive TME (**Figure 1**). In this review and for each CTX resistance mechanism, we report preclinical (based on HNSCC cell lines/xenograft) and clinical evidence of CTX resistance as well as ongoing clinical trials of CTX-based combined therapies to overcome CTX resistance (**Table 1**).

**Table 1:** Resistance mechanisms to chemotherapy (left), cetuximab (middle) and immunotherapy (right), described in head and neck squamous cell carcinoma.

	CHEMOTHERAPY		CETUXIMAB	
**Target alterations**	**DNA damage**		**EGFR and its ligands**	***EGFR alterations***
				Loss of PTPRS
				EGFR variant III
				Nuclear translocation
				SNP EGFR-K521
		DNA damage response effectors		EGFR G465R and concurrent EGFR G33S and EGFR N56K
		ERCC1 expression		***Competition with other ligands***
		ATR, WEE1 and PARP activation		Aberrant expression of TGF-a, TGF-b, EGF, HB-EGF, amphiregulin and heregulin
			**EGFR downstream effectors**	STAT3 activation by EGFR, JAK2 or a Src Kinase
				Src kinase activation
				RAS/MAPK pathway activation
				PI3K/Akt/mTOR pathway activation (*e.g. PTEN* mutation)
**Bypass pathway activation**	**Apoptosis evasion**	Survivin expression	**RTK activation**	MET, AXL, HER2, HER3, ROR2, IGF-1R and VEGFR
		Increased XIAP and TRAIL expression	**Apoptosis evasion**	Loss of the tumor suppressor gene *TP53*
			**Metabolism**	Hypoxia (*i.e.* HIF-1α overexpression)
**Epithelial-to-mesenchymal transition / cancer stem cells**	NEDD4 overexpression		Expression of lymphotoxin	
	miR-139-5p down-regulation		EGFR methylation	
	IL-6/STAT3 pathway activation		Secretion of CTX-containing extracellular vesicles	
	Increased expression of CD44 and Oct-4		Upregulation of EMT-related genes	
	Upregulation of ABC transporter genes		Loss of the tumor suppressor gene *SMAD4*	
	Increased ALDH1 expression			
**Epigenetic modifications**	Activation of miR-302		Altered expression of growth factor receptors and EMT-related genes by:	
	Increased FOXD2-AS1 expression		DNA methylation	
	Up-regulation of histone methyltransferase DOT1L		Histone modifications	
	*NEFL* promoter hypermethylation		Chromatin remodeling	
	Elevated expression of PAK2		Noncoding RNAs	
	miR-629-3p expression			
**TME**	Enhanced expression of miR-96-5p			
****	ALK1 activation		T regs and MDSC proliferation	
	SDF-1/CXCR4 expression		T cells exhaustion or impairment	
	Cancer associated fibroblasts (CAF) proliferation		Toll-like receptor 4 (TLR4) pathway activation	
			Cancer associated fibroblasts (CAF) proliferation	

IMMUNOTHERAPY	
**Intrinsic resistance**	***Tumor Immunogenicity and Antigen presentation***
	Selection of subclones lacking the expression of neoantigens
	downregulation of MHC class I (MHC-I)
	Loss of function of β2-microglobulin
	Alterations in STAT1
	***Oncogenic pathways***
	MAPK pathway
	WNT/β-catenin pathway
	PI3K pathway
	***Soluble molecules***
	Secretion of pro-tumoral cytokines IL-6, and IL-10
	IDO1 overexpression
	Secretion of immunosuppressive exosomes containing TGF-β, PD-1 and CTLA4
**Extrinsic resistance**	***Inhibitory checkpoint molecules***
	CTLA-4 expression in Treg TILs
	LAG-3
	TIM-3
	KIR2DL-1, KIR2D-2, KIR2D-3
	***Stimulatory agonist molecules***
	Costimulatory agonists: Ox40, 4-1BB, ICOS and CD40
	***Immunosuppressive cells***
	Myeloid derived suppressor cells
	Tregs
	Tumor-associated macrophages

For each resistance mechanism, preclinical and/or clinical evidence of their role in resistance as well as combined therapeutic strategies to overcome it are developed in the manuscript.

### Alterations in EGFR and Its ligands

Alterations of the antibody–receptor interactions can be induced by either alteration of the EGFR (82–92) or *via* competition with other EGFR ligands (87, 93–98).

#### EGFR Alterations

Several EGFR alterations have been reported in preclinical studies. First, loss of the EGFR phosphatase transmembrane protein tyrosine phosphatase RPTP sigma (PTPRS), which is known to directly interact, dephosphorylate, and inactivate EGFR (99), enhances EGFR-induced transformation and promotes EGFR/PI3K pathway activation, resulting in resistance to EGFR inhibition (88). Furthermore, constitutive activation of the EGFR, such as the EGFR variant III (EGFRvIII, activating mutation), results in activation of several downstream modulators (preferentially the PI3K pathway) and participates in increasing tumorigenicity and CTX resistance (92).

On the other hand, several CTX resistance mechanisms are based on the perturbation of CTX binding on EGFR. Indeed, contrary to the classic EGFR located on the plasma membrane, the nuclear EGFR (translocation mediated by the Src family kinases) cannot be targeted by CTX and, thus, functions as a transcription factor for several factors, inducing proliferation (cyclin D1, iNOS, B-myb, and aurora kinase A) (91). Moreover, the single nucleotide polymorphism encoding EGFRK521 (K-allele), which is expressed in >40% of HNSCC cases, has been shown to reduce stability of the EGFR and, thus, the affinity for CTX binding (85). Finally, the two concurrent, nonsynonymous missense G33S and N56K mutations in the extracellular domain of EGFR restrict adoption of a fully closed (tethered) and inactive EGFR conformation, thus, not permitting binding of CTX to the EGFR (82, 86).

Besides the preclinical evidence, clinical studies support some of the previously cited CTX-resistance mechanisms. Based on analysis of *n*=31 HNSCC (oral cavity) cases, Morris et al. find significant PTPRS loss or deletion in 32% of cases (88). They observed pathway activation (elevated levels of phospho-EGFR and phospho-AKT) in tumors with PTPRS deletion but not in tumors lacking PTPRS deletion. Smilek et al. show that a somatic EGFR mutation located in exon 19 may contribute to the limited clinical response to therapy with CTX plus radiotherapy (*n*=2/29 patients with advanced HNSCC) (86). Moreover, the high EGFRvIII expression, detected in 17%–42% of HNSCC tumors, was significantly and independently associated with shorter progression-free survival in patients with recurrent or metastatic HNSCC treated by CTX + Docetaxel (87, 92). For some authors, the role of this EGFR polymorphism in CTX resistance remains limited (100). The EGFR extracellular domain mutation G465R is reported to confer resistance to CTX by altering its binding to EGFR in a patient with a regional neck recurrence of an oral cavity HNSCC (83).

CTX-based combined therapy has been tested in the preclinical as well as clinical setting to overcome the previously cited resistance mechanisms. Dasatinib (BMS-354825), a tyrosine kinase inhibitor (TKI), limits the nuclear EGFR translocation (by blocking the Src family kinases), which leads to increased EGFR on the plasma membrane and restores sensitivity to CTX (90). Thus, Dasatinib is currently evaluated in combination with CTX in patients with recurrent HNSCC (NCT01488318, phase 2) (84) as well as in combination with CTX/cisplatin/RT (NCT00882583, phase 1). Interestingly, in the CTX + Dasatinib combination setting, patients with low serum IL6 have shown clinical benefit and improved OS (NCT01488318) (84).

#### Aberrant Expression of EGFR Ligands

The CTX-EGFR interactions are reduced in the context of competitive interaction with the overexpression of some ligands, such as TGF-α, TGF-β, EGF, HB-EGF, amphiregulin, and the aberrant HER3 ligand heregulin-expression (87, 93–98). Thus, this autocrine/paracrine growth factor production reduces CTX effectiveness in several HNSCC cell lines.

Indeed, based on the analysis of tumor biopsies from *n*=47 recurrent/metastatic HNSCC, the amphiregulin overexpression (representing 47% of cases) was a statistically significant prognostic factor of worse OS and progression-free survival (87). Yonesaka et al. report that *n*=2/28 HNSCC tumor samples that presented aberrant heregulin expression comparable to that of HNSCC CTX-resistant cell lines (FaDuCR cells) were resistant to CTX (94).

Interestingly, FaDuCR recovered the sensitivity to CTX in combination with Pertuzumab (anti-HER2 antibody) (94). Indeed, Pertuzumab prevents the binding of HER2 with its ligand (heregulin), avoiding the coupling of HER2/HER3, thus, resulting in the absence of the HER3-AKT pathway activation, which is responsible for inducing CTX resistance.

### Alterations of EGFR Downstream Signaling Effectors

Activation of downstream signaling effectors, such as STAT3 (signal transducer and activator of transcription 3) (77, 84, 101–106), Src Kinases (64, 107, 108), RAS/MAPK pathways (86, 96, 109–115), and PI3K/Akt/mTOR pathway (102, 105, 116–127) could induce CTX resistance independently of the EGFR-ligand activation.

#### STAT3 Activation

STAT3, a member of the STAT family of transcription factors, is considered as an oncogene activated in several cancers, including HNSCC (128). Its activation could be driven by EGFR as well as in an independent EGFR way by another growth factor receptor, the Janus kinase 2 (JAK2) or by the Src kinase family. Furthermore, loss of the PTPRS tumor suppressor gene that dephosphorylates STAT3 may lead to permanent activation of STAT3. Several studies report that hyperactivation of STAT3 is implicated in CTX treatment resistance.

Indeed, several HNSCC cells lines that develop acquired resistance to CTX are characterized by increased total STAT3 expression (77). The role of STAT3 in HNSCC cell CTX that acquires resistance is supported by recovering increased sensitivity to CTX (greater antiproliferative effects and cytotoxicity) when STAT3 is knocked down (104) or by blocking JAK2–STAT3 signaling (using miR-204) (101). Moreover, analysis of *n*=22 samples from patients with HNSCC tumors that recurred following CTX treatment finds increased phosphorylated STAT3 (103).

Regarding CTX-based combinations, guggulsterone, a natural compound contained in the Commiphora mukul plant resin used in Indian ayurvedic medicine and considered as an anti-STAT3 agent, enhances the efficacy of CTX when combined with CTX (106). Moreover, the combination of CTX + JAK2 inhibitor (miR-204) inhibits STAT3 activation, resulting in inhibition of angiogenesis and promotion of *in vivo* CTX sensitivity (101). Given that STAT3 may be activated by the Src kinase family, Dasatinib (SRC inhibitor), which is tested in combination with CTX in phase-2 (84) and phase-1 clinical trials (NCT01488318 and NCT00882583, respectively), could provide some insight about the utility of the STAT3 inhibition in overcoming CTX resistance.

#### Activation of Src Kinases

Src family kinases are frequently overexpressed and/or activated in several cancers, including those arising in the head and neck (85). These nonreceptor protein tyrosine kinases play critical roles in signaling pathways, regulating cell division, motility, adhesion, angiogenesis, and survival (129). Thus, activation of Src kinases could be involved in proliferation/migration/invasion of cancer cells as well as in treatment resistance.

Based on gene expression profiles of CTX-resistant OSCC cells as well as of publicly available data sets, Uzawa et al. identify a 12-gene expression signature of CTX resistance, including the urokinase-type plasminogen activator receptor (uPAR) (107). They show that CTX resistance could be mediated by uPAR upregulation. Indeed, through the uPAR/integrin β1/Src/FAK signal circuit, the uPAR upregulation activates ERK1/2 phosphorylation to maintain cell proliferation/invasion resulting in CTX resistance *in vitro* and *in vivo* even in the absence of EGFR overexpression or acquired activating mutations. Src kinases could also induce CTX resistance by EGFR-ligand independent transactivation (cell-substratum adhesion), which phosphorylates ErbB3 to form a heterodimer complex, inducing proliferation *via* AKT (108).

Based on previous evidence, the CTX-based combination with the Src inhibitor-1 or resveratrol (uPAR inhibitor) are shown to overcome CTX resistance *in vitro* and *in vivo* (tumor growth suppression and uPAR downstream protein downregulation), respectively.

#### Activation of the RAS/MAPK Pathway

The family of mitogen-activated protein kinases (MAPK) are a family of serin-threonin kinases implicated in the regulation of the majority of physiological cellular processes, including proliferation, differentiation, and apoptosis in response to changes in the cellular environment (130). In particular, the Ras/Raf/MEK/ERK1/2 (extracellular signal-regulated protein kinases) cascade is the MAPK signaling cascade most frequently associated with carcinogenesis of several cancer types (131). Regarding HNSCC, this signaling cascade can be activated by several tyrosine kinase receptors, such as EGFR, as well as independently of them by alterations of the Ras/Raf oncogenes. This highlights the variety of CTX-resistance mechanisms involving this signaling cascade.

First, the MAPK signaling pathway activation related to the HNSCC CTX resistance could involve a RAS-activating mutation (G12V HRAS) (113). The restored sensitivity to CTX by silencing H-Ras in H-Ras mutant HNSCC cell lines reinforces this observation (132). However, activation of the RAS/MAPK pathway even in the absence of constitutive gene mutations could lead to CTX resistance (111). Indeed, overexpression the K-Ras, H-Ras, and N-Ras proteins (96) leads to CTX resistance. Furthermore, dysregulation of the regulating proteins of the RAS/MAPK pathway could also contribute to CTX resistance as supported by the low expression of DUSP5 and DUSP6 (negative regulators of ERK1/2) and upregulation of AURKB (100) and AURKA (114), which are key regulators in mitosis.

In the clinical setting, Braig et al. show that acquisition of RAS mutant clones (KRAS G12S, G13C; NRAS Q61K, NRAS A146P; HRAS G13R) correlates significantly with clinical resistance to CTX in a cohort of *n*=20 patients treated by CTX/platinum/5-fluorouracil treatment with monitoring of the circulating tumoral DNA (ctDNA) (110). The role of the KRAS p.Gly12Val mutation in CTX resistance previously found *in vitro*, is also demonstrated in only one patient carrying this mutation (among the *n*=29 studied) associated with an absence of response to treatment (86). Rampias et al. confirm that the HRAS mutation (*n*=7/50 patients with HNSCC) is associated with *de novo* resistance to CTX-based therapy (113). Overall, Bossi et al.’s observations (cohort of *n*=40 recurrent/metastatic HNSCC) are in accordance with others and show that overactivation of the RAS pathway leads to CTX/platinum resistance (111).

On the basis of their results, especially the interesting observation of the crosstalk between the RAS/RAF/MAPK and PI3K/AKT pathways, Rampias et al. tested the combination of CTX + a PI3K inhibitor (LY294002) in an H-Ras mutated cell line and found a marked reduction of their viability (113). Apigenin, an ERK 1/2 inhibitor, in combination with CTX resulted in a significant decrease of HNSCC CTX-resistant cell survival (112). Interestingly, the combination of CTX + tipifarnib (farnesyltransferase inhibitor) showed enhancement of the tipifarnib antitumor effect through concomitant ERK inhibition *in vitro* and *in vivo* (109). Finally, a combination of CTX with inhibition of the ERK upregulators, i.e., aurora kinase knockdown (siRNA) and inhibitor (the pan aurora kinase inhibitor R763), showed inhibition of proliferation and increased apoptosis in HNSCC cells lines (112, 114, 115).

#### PI3K/Akt/mTOR Pathway

The phosphatidylinositol-3-kinase (PI3K)/Akt and the mammalian target of rapamycin (mTOR) signaling pathways are involved in several physiological as well as pathological cellular processes, including proliferation, differentiation, survival, and motility (133). In HNSCC, PI3K/AKT/mTOR signaling is active in more than 90% of HNSCC as a result of EGFR activation, PI3K overexpression, phosphatidylinositol-4,5-bisphosphate 3-kinase catalytic subunit alpha (PI3KCA) mutations/amplifications, and PTEN mutation (116, 134). Activated PI3K/AKT/mTOR signaling is related to radiotherapy and cytostatic drug resistance, likely through enhanced DNA-repair mechanisms.

Several genetic alterations causing PI3K/AKT/mTOR activation, such as activating mutations in the oncogene PI3KCA or inactivating mutations in the tumor suppressor protein PTEN, are driving CTX resistance in different HNSCC cell lines (125, 127). Indeed, Izumi et al. show that loss of PTEN conferred independence from EGFR activity and resistance to EGFR inhibition by CTX in terms of downstream signaling, proliferation, and tumor growth both *in vitro* and in *in vivo* xenograft models (119).

Moreover, Eze et al. recently reported the analysis of PTEN and PIK3CA expression in samples from patients with recurrent or metastatic HNSCC enrolled in two trials of cetuximab-based therapy (*n*=48 patients in the E5397 trial and *n*=37 in the NCI-8070 trial) (117). Patients with low PTEN expression had significantly worse survival.

Thus, CTX-based combined therapy has been realized using ATP-competitive PI3K inhibitors as well as mTOR inhibitors (Rapamycin and analogues). Regarding ATP-competitive PI3K inhibitors, the CTX combination with Buparlisib or BKM120 demonstrates the highest antiproliferative effect and inhibition of PI3K/protein kinase B, AKT/mTOR signaling pathways *in vitro* (122) and *in vivo* (121). The BYL719 (PI3Kα specific inhibitor), namely Alpelisib (123) and the Copanlisib (highly selective, pan-class I PI3K inhibitors) (120), are shown to improve CTX-induced tumor inhibition in HNSCC CTX-resistant cell lines and PDX. Interestingly, the combination of CTX plus the PKI-587 (PI3K/mTOR inhibitor), namely Gedatolisib, which restored sensitivity to CTX in resistant HNSCC cell lines and xenografts (124), is found to have a greater synergistic enhancement of the CTX effectiveness, especially in basal-like HNSCC cells with mutated CDKN2A (118). Regarding mTOR inhibitors, Rapamycin (Rad001) (126) and Temsirolimus (105) show improving CTX antiproliferative effects in xenografts.

Interestingly, combinations of CTX with PI3K/Akt/mTOR inhibitors are widely investigated in clinical trials. Regarding ATP-competitive PI3K inhibitors, there are several phase-1 and -2 trials enrolling patients with HNSCC to be treated by CTX + Buparlisib (BKM 120) (NCT01816984, phases 1 and 2), Alpelisib (BYL719) (NCT01602315, phases 1b and 2) (126), and Copanlisib (NCT02822482, phases 1b and 2, COPAN-ORL06, specifically for patients harboring a PI3KCA mutation/amplification and/or a PTEN loss). PX-866, a noncompetitive PI3K inhibitor, was also tested in combination with CTX (NCT01252628, phases 1 and 2). Analogues of the rapamycin, temsirolimus (NCT01256385, phase 2, MAESTRO HN) and everolimus (NCT01637194, phase 1; NCT01283334, phases 1 and 2) (57) have already brought some interesting results to overcome CTX resistance.

### Bypass-Pathway Activation

Another resistance mechanism involves the abnormal activation of parallel signaling pathways to counteract the blockade of the EGFR signaling by CTX. Thus, cancer cell survival is ensured by increased expression/activation of alternative receptor tyrosine kinases (RTK) (50, 77, 79, 123, 135–150), ensuring the activation of several parallel pathways, including the VEGF pathway (72, 151–153).

#### Receptor Tyrosine Kinases

Among the growth-factor receptor family, RTK are transmembrane receptors implicated in several physiological as well as pathological (oncogenesis) processes (154). The binding ligand-extracellular domain induces receptor dimerization, activation of the intrinsic tyrosine-kinase activity of the RTK, and activation of downstream signaling cascades implicated in cell proliferation, differentiation, motility, survival, and cell–cell communication (155). Thus, activation of these RTKs is a mechanism of resistance to CTX during HNSCC treatment (50, 77, 79, 123, 135–150).

Indeed, the increased expression and activation of RTK, such as MET, AXL, HER2, HER3, and ROR2, are reported in several CTX-resistant cell lines (148) as well as *in vivo* (PDX). For example, MET/HGF (146) as well as AXL (123, 139, 148) overexpression and activation stimulate cell proliferation despite CTX treatment *in vitro* and *in vivo*, especially through MAPK downstream signaling. Recently, McDaniel et al. investigated the AXL-mediated CTX-resistance mechanisms in HNSCC and report that the tyrosine 821 of AXL mediates resistance to CTX by activation of c-ABL (oncoprotein) (156).

Other ErbB family members, ErbB2 (HER2) (102) and ErbB3 (HER3), could also be implicated in CTX resistance. Indeed, Yonesaka et al. report the persistence of ERK 1/2 signaling caused by the permanent activation of ErbB2 signaling (amplification of the receptor ErbB2 or upregulation of the ligand heregulin) induces CTX resistance in HNSCC cell lines (141). The restoration of CTX sensitivity through inhibition of ErbB2 or disruption of ErbB2/ErbB3 heterodimerization reinforce their observations. On the other hand, this heterodimerization also highlights the role of HER3 activation in resistance to CTX treatment of some HNSCC cell lines (135). The permanent activation of ErbB3/Akt signaling could be caused by an autocrine neuregulin expression (autocrine loop) as well as by aberrant HER3 ligand heregulin expression (94). Furthermore, increased activity of the IGF1R signaling pathways has been reported in several CTX-resistant HNSCC cell lines (136, 137, 143). IGF1R and HER3 activations with partial EGFR persistent activity are intertwined during CTX resistance as supported by the ability of a multitarget mAb mixture against EGFR, HER3, and IGF1R to overcome CTX resistance.

These preclinical observations are supported by clinical evidence. Indeed, in a retrospective cohort of *n*=57 patients with recurrent/metastatic HNSCC, patients who presented HGF/MET pathway overexpression and activation had worse prognoses (138). Moreover, Chung et al. report the case of a patient with recurrent HNSCC who presented an interesting response to AMG-479 (a monoclonal antibody against IGF-1R) after CTX resistance. Tumor sample analysis suggests the potential benefit of a combined therapy using AMG-479 plus CTX (142).

To overcome CTX-resistance, several combined therapies using RTK inhibitors have been tested. The BET inhibitor JQ1, which binds preferentially to the bromodomains of BRD4, abrogates the expression of the alternative RTK (HER3 and AXL), resulting in significantly delayed acquired resistance in two PDX models of HNSCC (148). Combined with CTX, MET inhibitor PHA-665752 is also shown to restore CTX sensitivity *in vitro* and *in vivo*, especially by decreasing akt and ERK1/2 phosphorylation (146, 147). Inhibition of the AXL receptor is explored by using imatinib (which targets c-Abl) in CTX-resistant HNSCC PDX (156). This led to complete tumor regression and a prolonged effect (no recurrence up to 3 months after cessation of treatment). Moreover, the Lida et al. experiment brings general support to the implication of several ErbB family members in CTX resistance. They find that the pan-HER mixture of six antibodies targeting EGFR, HER2, and HER3 decreases HER family receptors in acquired CTX-resistant HNSCC cells lines and overcomes CTX resistance in PDX (98). More precisely, a dual EGFR/HER2 inhibitor with CTX plus Afatinib shows significantly improved tumor volume reduction in CTX-resistant xenografts compared with either agent alone in monotherapy (140). ErbB3 inhibition has also been realized *in vitro* and *in vivo* using MM-121 (97, 113) as well as CDX-3379 (ErbB3-specific blocking antibody) (93). These combinations inhibit proliferation through inhibition of PI3K/Akt and ERK signaling pathways. When combined with CTX, the anti-IGF-IR antibody (IMC-A12) A12 provides important inhibitions of cell proliferation and migration *in vitro* and *in vivo* (regression of tongue cancer cell xenografts) (144). Although the rationale of dual VEGF and EGFR inhibition is proposed in several other cancers (157), Argiris et al. show that combined targeting of EGFR with CTX and VEGF with bevacizumab enhances growth inhibition both *in vitro* and *in vivo* (153).

Given that ErbB3 activation induced by heregulin is previously described as a CTX-resistance mechanism, the combination of Patritumab (U3-1287), an anti-HER3 monoclonal antibody, and CTX with platinum-based therapy was evaluated in a randomized, double-blind, phase-II study of first-line treatment of patients with recurrent or metastatic HNSCC (NCT02633800) (158). Although tolerable, the combination Patritumab + CTX + platinum was not superior to CTX + platinum. Based on the previous rationale as well as on a phase-I study (149), Deeken et al. evaluate the combination of lapatinib (which blocks both EGFR and ErbB2) plus CTX (NCT01184482) in patients with advanced solid malignant tumors, including HNSCC. Results were interesting with an overall response rate of 17% and a clinical benefit rate of 67%.

CDX-3379, an anti-ErbB3 monoclonal antibody, has been recently reported to inhibit tumor ErbB3 phosphorylation in HNSCC and induce measurable tumor regression and was well tolerated (93). Thus, a phase-2 clinical trial (NCT03254927) is ongoing and aims to determine the clinical benefit, safety, and tolerability of combining CDX-3379 and CTX in patients with advanced HNSCC who have previously received CTX and progressed.

IGF-1R inhibitors are also widely explored in combination with CTX for recurrent/metastatic HNSCC. Glisson et al. as well as Ferrarotto et al. report no improvement of progression-free survival and OS using the Cixutumumab + CTX compared with CTX alone (159, 160). The OSI-906, a dual kinase inhibitor of both IGF-1R and insulin receptor was evaluated in combination with CTX among patients with HNSCC (NCT01427205, phase 2), but results are not available. More recently, the combination of CTX plus the anti-IGF-1R antibody A12 (IMC-A12) was evaluated in the neoadjuvant setting for patients with HNSCC NCT00957853 (Phase 2).

Finally, VEGF inhibitors combined with CTX have been also investigated. Although some results are not available (NCT00906360, phase 1, CTX + Sunitinib), others are contradictory. Indeed, although some trials report that bevacizumab + cisplatin + CTX + intensity-modulated radiation therapy (IMRT) in locally advanced HNSCC is associated with favorable efficacy outcomes (NCT00968435, phase 2), Argiris et al. find that adding bevacizumab increases toxicity without apparent improvement in efficacy (NCT00703976, phase 2) (151). Thus, the potential clinical benefit of combined EGFR–VEGF targeting is not clearly established.

#### Other Signal Transducers

Several proteins involved in classic cancer pathways, such as proliferation, apoptosis, invasion, and metastasis, could be altered and implicated in CTX resistance during HNSCC treatment (64, 115, 161–165).

Among all somatic genomic alterations in HNSCC, the tumor suppressor gene *TP53* is the most frequent (166), highlighting its importance in carcinogenesis and progression. Indeed, although the tumor suppressor protein p53 has a critical role in cell cycle arrest, apoptosis, and senescence, loss of its function is linked to disease progression and treatment response (64). Regarding CTX, comparative analysis of sensitive vs. CTX-resistant HNSCC cells reveals the central role of the loss of p53 in the development of acquired resistance to CTX (163).

The precise role of hypoxia in acquired resistance to cetuximab is not clearly established, and further studies are needed. Indeed, Boeckx et al. find that the sensitivity to CTX is not altered but increased in HNSCC cells exposed to prolonged hypoxia (164). On the other hand, Lu et al. report that HNSCC cells with acquired CTX resistance express a high level of the alpha subunit of the hypoxia-inducible factor-1 (HIF-1α) and are highly glycolytic (aerobic glycolysis, i.e., the Warburg effect). Furthermore, the experimental overexpression of HIF-1α confers resistance to CTX as well as abolishes CTX-mediated radiosensitization in HNSCC cells (161).

These preclinical observations have caused Lu et al. to explore the inhibition of hypoxia and its relationship with CTX efficacy. Downregulation of HIF-1α by siRNA or a small molecule inhibitor (1-methyl 1, 9 PA) enhances response of CTX-resistant HNSCC cells to CTX plus radiotherapy (161). Finally, Lu et al. confirm that CTX inhibits HNSCC cell proliferation through inhibition of glycolysis and that the combination of CTX + oxamate (inhibition of LDH-A, an enzyme catalyzing the conversion of pyruvate to lactate in anaerobic conditions) improves the therapeutic effect of CTX in cancer cells (162). As a continuation of their work on the role of hypoxia and glycolysis during HNSCC treatment, Lu et al. explore the role in CTX resistance of the mitochondrial enzyme pyruvate dehydrogenase kinase-1 (PDK1), known to allow the switching glucose metabolism toward aerobic glycolysis in cancer cells (165). They found that the combination of CTX plus PDK1 knockdown (siRNA) or with pharmacological inhibition of PDK1 with dichloroacetic acid (DCA) overcomes CTX-resistance *in vitro* and *in vivo* (xenografts) thanks to the overproduction of reactive oxygen species (ROS) and the subsequent apoptosis.

### Epithelial-to-Mesenchymal Transition

The importance of EMT in human disease, especially in carcinogenesis, has been reviewed elsewhere (167). The EMT can be considered as a continuum of multiple and dynamic transitional states whereby cells exhibit epithelial, intermediate, and mesenchymal phenotypes. Regarding HNSCC, acquisition of an EMT phenotype (modulation of cell polarity and adhesion) by cancer cells is involved in disease progression as well as in CTX resistance (168–179).

Indeed, several authors report that HNSCC cells exhibiting a mesenchymal-like phenotype are resistant to CTX treatment *in vitro* and *in vivo* (xenografts) (174, 176, 177). Several potential mechanisms implicated in this EMT-induced CTX resistance are observed, such as (i) expression of lymphotoxin-b; (ii) methylation of EGFR that promotes the EGFR ligand-binding ability and dimerization (EGFR persistent activity) (169); (iii) secretion of CTX-containing extracellular vesicles, which lead to cancer cell protection (179); (iv) upregulation of EMT-related genes (133), especially by epigenetic regulation (170, 180); and (v) loss of the tumor suppressor gene SMAD4, which induces JNK and MAPK pathway activation (172, 173). Indeed, Ozawa et al. find that SMAD4 loss is associated with CTX resistance and poor survival in HPV-negative patients (cohort of *n*=130 newly diagnosed and *n*=43 patients with recurrent HNSCC) (172). Thus, Ozawa et al. tested the combination of CTX + JNK inhibitor (SP600125) or MAPK/MEK inhibitor (U0126) and show that it contributes to overcome CTX resistance *in vitro*.

Moreover, the development of CTX resistance could also be accompanied by increasing hedgehog pathway transcription factor expression *in vitro* (175). Thus, Keysar et al. tested the combination of CTX and IPI-926 (hedgehog pathway inhibitor) in four different PDX models. This combination forced tumor cells into an EGFR-dependent state and blocked tumor recurrences.

### Epigenetic Modifications

Epigenetic alterations, including DNA methylation, histone modifications, chromatin remodeling, and noncoding RNAs, are frequently involved in head and neck carcinogenesis, tumor progression, and resistance to therapy (137), especially to CTX (75).

As previously described, Kagohara et al. report that genes associated with CTX resistance in HNSCC cell lines, including TFAP2A, which regulates growth factor receptors and EMT, are epigenetically regulated (170). Stein-O’Brien et al. show that FGFR1 demethylation is associated with CTX resistance and this type of epigenetic alteration might stabilize the resistant phenotype (171). Interestingly, Shimizu et al. recently reported that nicotine (one of the main tobacco components) contributes to CTX resistance *in vitro* as well as *in vivo* (xenografts) (181). Indeed, they show that nicotine induces, through the nicotinic acetylcholine receptor, both EGFR phosphorylation (and the subsequent Akt and mTOR downstream cascade activation) and nuclear translocation of the phosphorylated EGFR.

### Establishment of an Immunosuppressive TME

**Figure 2:**
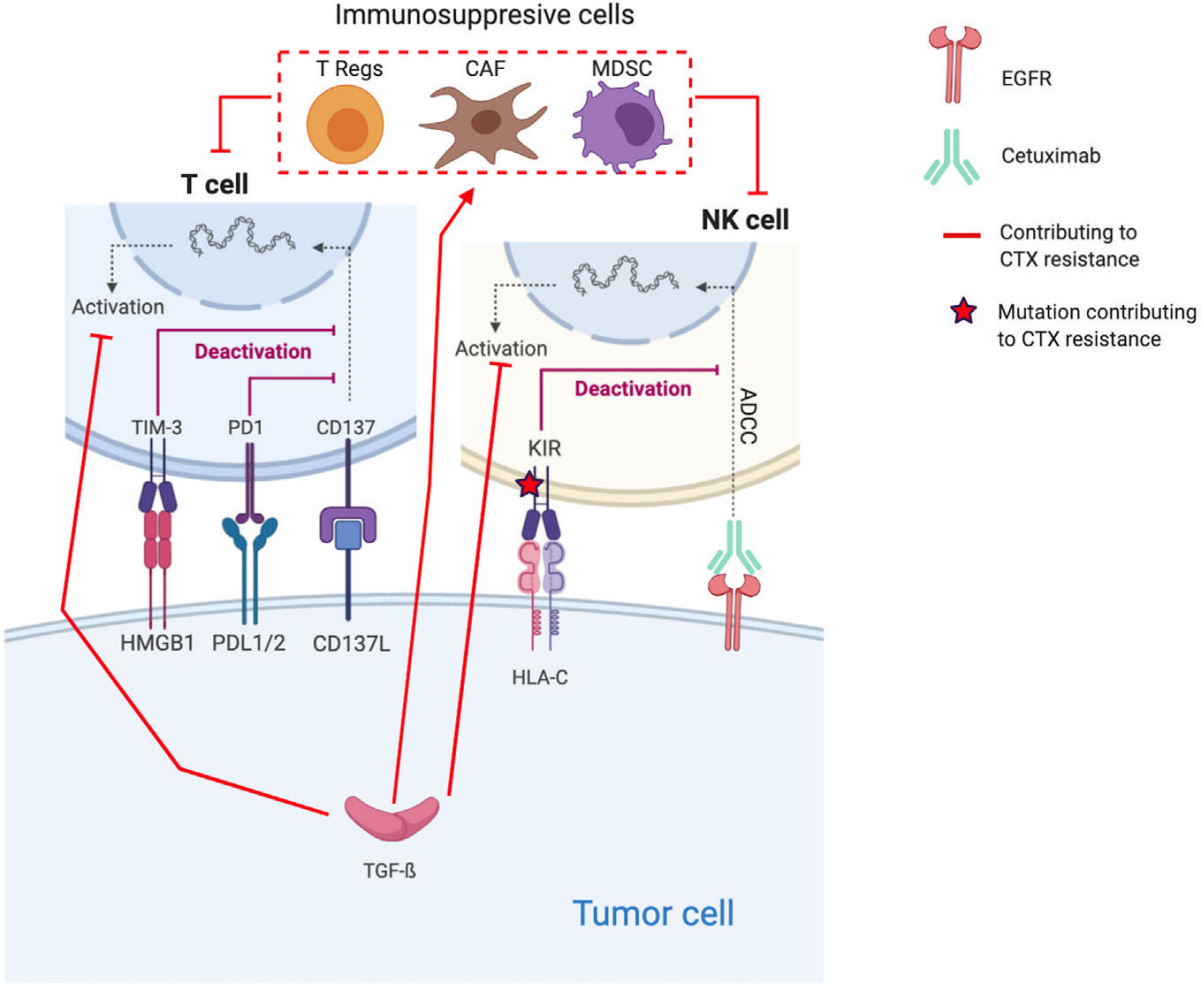
Molecular mechanisms contributing to Cetuximab resistance through the establishment of an immunosuppressive TME. Red lines and arrows show mechanisms contributing to Cetuximab resistance, and green lines and arrows show mechanisms contributing to Cetuximab sensitivity. (EGFR, Epidermal Growth Factor Receptor; NK, Natural Killer; PD1, Programmed death 1; PDL1, Programmed Death Ligand 1; KIR, Killer Immunoglobulin-like Receptor; ADCC, Antibody-dependent cellular cytotoxicity; HLA-C, Human leukocyte antigen-C; TGF, transforming growth factor; Treg, regulatory T-cells; MDSC, Myeloid-derived suppressor cells).

In addition to all the previously cited intrinsic resistance mechanisms, there are also extrinsic resistance mechanisms, i.e., involving the TME (182). These mechanisms encompass, in fact, the cancer cell–TME crosstalk, contributing to CTX resistance (40, 75). In HNSCC, establishment of an immunosuppressive microenvironment is an important resistance mechanism to treatment, especially to CTX (6, 70, 183, 184) (**Figure 2**).

#### Regulatory T Cells (Tregs) and Myeloid-Derived Suppressor Cells (MDSC) Proliferation

Proliferation of immunosuppressive cells, such T regs and MDSC, in response to CTX treatment is one of the key resistance mechanisms (185–188). Indeed, several coculture experiments show that CTX expands CTLA-4+FOXP3+ Tregs in part by inducing dendritic cell maturation. These Tregs lead to CTX resistance by suppressing the CTX-mediated ADCC (cytolytic functions of NK cells) (188). Based on the analysis of blood samples from CTX-treated patients with locally advanced/metastatic (stage III/IV) HNSCC (*n*=22 patients, NCT 00226239 and *n*=18, NCT 01218048), Jie et al. confirm their *in vitro* observations (188). Indeed, they find that CTX increased the frequency of intratumoral Tregs expressing the inhibitory checkpoint cytotoxic T-lymphocyte-associated protein 4 (CTLA-4), which is known to inhibit T cell activation (6). Thus, Tregs suppress the CTX-mediated ADCC. Their presence is correlated with poor clinical outcomes in these cohorts. Based on these results, Jie et al. tested CTX in combination with ipilimumab, a monoclonal antibody that also induces NK cell–mediated ADCC. Ipilimumab treatment enhanced the CTX-mediated ADCC by eliminating Tregs (targeting CTLA-4), allowing effector T cell activation and restoration of the cytolytic functions of NK cells (*n*=6 HNSCC tumors) (188).

Furthermore, Shayan et al. hypothesized that the combination of CTX plus motolimod, a small-molecule TLR8 agonist that can activate monocytes, DCs, and NK cells (189), might enhance T cell stimulation and CTX effects (187). They find that the TLR8 stimulation through motolimod skewed monocytes toward an antitumor M1 phenotype and reversed MDSC suppression of T cell proliferation. These *in vitro* observations are confirmed in patients. Indeed, in a phase-Ib trial (NCT02124850) enrolling *n*=14 patients with previously untreated stage-III/IV HNSCC, Shayan et al. tested the combination of CTX plus motolimod (formerly VTX-2337) (187). The combination reversed MDSC-induced immunosuppression and improved antitumor immunity with increased circulating tumor antigen-specific T cells (EGFR specific) and increased the number and function of tumor-infiltrating CD8 T cells. These encouraging results are confirmed in another phase-Ib study (*n*=13 patients with recurrent/metastatic HNSCC, NCT01334177) demonstrating the significant increasing antitumor activity of this combination (increased plasma cytokines and activated circulating NK cells). To finish, the potential benefit of adding motolimod to the standard EXTREME regimen (CTX + platinum + fluorouracil) was evaluated in a phase-2 trial enrolling *n*=195 patients with recurrent/metastatic HNSCC (NCT01836029) (185). Ferris et al. find that the combination fails to prove a benefit for survival when considering the intent-to-treat population, but significant benefits are observed when considering only the selected subgroup of patients with HPV-positive tumors and injection site reactions.

#### T Cell Exhaustion/Impairment

HNSCCs are among the most immune-infiltrated cancers, and several mechanisms are implemented by tumor cells to escape to the host immune defense system (190, 191). The immune-modulatory effect of CTX treatment, in particular ADCC, might be inhibited by cancer cells through several mechanisms inducing T cell exhaustion/impairment and all CTX resistance (67, 97, 192–204). Indeed, to counteract the antitumor activity of CTX, tumor cells express TGF-β, which inhibits the expression of cytotoxic effector molecules in immune cells (Apo2L/TRAIL, CD95L/FasL, granzyme B, and IFN-γ) and suppresses their ability to induce cetuximab-mediated ADCC (97).

Moreover, in a cohort of *n*=18 patients with stage-III/IV HNSCC treated by CTX alone (NCT 01218048), Jie et al. find that the increased frequency of PD-1+ and TIM-3+ tumor-infiltrating lymphocytes (TILs) during CTX treatment inversely correlates with objective response (200). Besides PD-1 and TIM immune checkpoint (ICP) receptors, KIR, the ICP on NK cells that modulate their activation, is also indirectly implicated in CTX resistance. Indeed, Faden et al. report a statistically significant increase of missense mutations and loss of heterozygosity in HLA-C (the ligand for KIR) in patients not responding to CTX compared with responders (196).

In accordance with the previously cited *in vitro* observations, several CTX combinations have been tested in the preclinic as well as clinic setting.

For example, Bedi et al. explored the combined effect of CTX and TGF-β blocking *in vivo* (PDX). Although CTX alone forced the selection of resistant clones, i.e., TGF-β–overexpressing tumor cells, the combined treatment prevented it and induced complete tumor regression (97). In the same way, Faden et al. observed increased HNSCC cell killing when combining CTX and lirilumab, a monoclonal antibody that blocks NK inhibitory KIR signaling (196). Moreover, the upregulation of CD137 or 4-1BB (a member of the TNF-receptor superfamily, which is broadly induced and activated on several effective immune cells) was corelated to clinical response to neoadjuvant CTX (NCT01218048). Srivastava et al. tested the effect on several HNSCC cell lines of the combination CTX plus urelumab (BMS-663513, CD137-agonist monoclonal antibody) (201). This combination enhanced the CTX-mediated ADCC as supported by the increased NK-cell survival, DC maturation, and tumor antigen cross-presentation. Regarding other ICP inhibitory signals, Concha-Benavente et al. explored several HNSCC cell lines and found that the programmed death ligand-1 (PD-L1), which limits the function of activated T lymphocytes when they interact with the ICP receptor programmed death-1 (PD-1), is expressed by tumor cells in an EGFR- and JAK2/STAT1-dependent manner (159). Thus, they tested the combination of CTX and JAK2 inhibition. They found that JAK2 inhibition prevented tumor PD-L1 expression and that the combination enhanced the CTX NK-mediated killing *via* ADCC against PD-L1+ HNSCC cells.

The better comprehension of immune evasion mechanisms as well as of the immune-modulatory effect of CTX, i.e., CTX-mediated ADCC, brings evidence to support the evaluation of combined approaches with ICP inhibitors in both locally advanced and recurrent/metastatic HNSCC (6, 67, 192). Among the tested agents, inhibition of the PD-1/PD-L1 synapse is widely explored. The anti PD-1 Nivolumab, for which efficacy and safety prior to CTX in HNSCC has been recently reported (CheckMate 141) (205), is actually tested in combination with CTX in phase-1 and -2 trials for patients with recurrent/metastatic HNSCC (NCT03370276). Sacco et al. recently reported some preliminary results of the first trial evaluating the antitumor activity of anti-PD-1 Pembrolizumab combined with CTX in *n*=33 patients with platinum-refractory/ineligible, recurrent/metastatic HNSCC (NCT03082534, phase 2) (197). Results are promising with an observed 41% response rate. Regarding anti PD-L1, Durvalumab combined with CTX and radiotherapy is currently tested in a phase-I/II clinical trial (NCT03051906, DUCRO) (193). Based on previous safety studies (154, 163, 167), several clinical trials are testing the anti PD-L1 Avelumab in combination with CTX (NCT03494322, phase 2 EACH) (206) +/- radiotherapy (NCT02999087 phase 3 REACH) or Palbociclib (a selective CDK4/6 inhibitor) (NCT03498378 phase 1 and NCT02101034 phase 2) (195). Other interesting combinations involve the 4-1BB agonist Urelumab (NCT02110082) (201), the anti-CTLA-4 monoclonal antibody ipilimumab (NCT01935921, phase 1) (202) and Monalizumab (anti NKG2A receptors expressed on TIL-CD8+ and NK cells) (NCT02643550) (199).

#### Toll-Like Receptor 4 (TLR4) Pathway Activation

TLR4 is an innate immune receptor involved in defense against microbial agents by recognizing inflammation-associated microbial ligands (such as lipopolysaccharide) and promoting the activity of innate immune cells (207). TLR4 are also expressed by tumor cells, and the role of the TLR4 signaling pathway in the TME has been reviewed elsewhere (208). Unfortunately, by inducing immunosuppressive cytokines, apoptosis resistance, and EMT, the TLR4 signaling pathway can promote cancer cells’ immune escape in several cancer types (including lung, pancreas, and ovarian cancers, HNSCC) as well as resistance to therapy (paclitaxel in ovarian cancer) (209–212).

Indeed, Ju et al. recently reported that the crosstalk between the EGFR and TLR4 pathways could participate in CTX resistance *in vitro* and *in vivo* (xenograft) (213). They found that EGFR inhibition led to decreased MyD88 degradation, and thus, MyD88 could activate TLR4 (receptor homodimerization). TLR4 activation induced activation of NF-κB and MAPK signaling pathways, resulting in the release of proinflammatory cytokines (TNF-α, iNOS, COX2, PGE2, NO) favoring EGFR permanent activation as well as the release of anti-apoptosis proteins (Bcl-2, Bcl-xl) allowing tumor cell survival. Overall, the TLR4 signaling pathway leads to CTX-resistance. Thus, Ju et al. tested the combination of CTX and a TLR4 inhibitor (TAK242). They find that this combination overcomes acquired CTX resistance *in vivo*, in particular by decreasing the secretion of pro-inflammatory cytokines (TNF-α, PGE2, and NO) (175).

#### CAF Proliferation

Among the different components of the HNSCC TME, CAFs are among the most critical elements contributing to proliferation, invasion, and metastasis (214), in particular by altering the antitumor immune response (215, 216). Furthermore, CAFs have been shown to contribute to drug resistance in HNSCC (e.g., platinum and CTX) (42, 217–219).

Indeed, during CTX treatment, CAFs, especially those activated by TGF-β (218), participate in resistance by secreting immunosuppressive factors, such as IL-6, HGF, and metalloproteinases (219). Thus, co-inhibition of TGF-β and HNSCC cells by the combination of CTX + SIS3 (an inhibitor of the TGF-β pathway), delayed tumor progression and lowered tumor volume/weight (HNSCC PDX) (218). Johansson et al. also tested the combined effect of CTX + MMP inhibitor III (inhibiting MMP-1, -2, -3, -7, and -13), which significantly reduced the protective effect of CAFs (219).

## Immunotherapy in HNSCC

HNSCC is among the most inflamed, immune-infiltrated cancers, especially with CD8+ TILs and NK cells (183). A high genetic instability and somatic mutation rate is often observed (220, 221) (about 180 somatic mutations per mega base), in either HPV positive or negative tumors (222, 223). Immunogenicity of HNSCC can result from the overexpressed but nonmutated native proteins that have escaped central tolerance, neo-antigens derived from mutated proteins (224), or HPV-induced viral antigens (e.g., oncogenic drivers E6 and E7) (225). The microenvironment can also vary in terms of intensity or constitution depending on carcinogens or localization (226).

Genetic and epigenetic alterations in cancer cells create a vast array of neoepitopes potentially recognizable by the immune system. However, a key feature of malignant cells is their ability to escape recognition by the immune system, and the dysregulation of immune checkpoints, such as PD-L1, in tumors appears to be a major immune-resistance mechanism affecting T cell response. It was, hence, shown that the reversal of the anergic state of T lymphocytes is possible *via* the blockade of coinhibitory signals (227), and research initially focused mainly on immune checkpoint inhibitors (ICI) of the PD-L1/PD-1 axis. These antibodies (Abs) have since completely transformed the treatment of R/M HNSCC.

The expression of PDL1 is positive in most cases, estimated at almost 60%–70% with a higher expression in HPV+ compared to HPV- tumors (228, 229). It results either from an immune-adaptive phenomenon induced by IFNγ or from intrinsic oncogenic events, such as the mutation/deletion of the PTEN suppressor gene or the deregulation of the AKT/mTOR, NF-kB and mitogen-activated protein kinase (MAPK) pathways (230). PD-1 binding with PD-L1/PD-L2 causes immunosuppression *via* reduced t cell receptor (TCR) signaling, reduced cytokine production, reduced target cell lysis, altered lymphocyte motility, and metabolic reprogramming (231).

After showing antitumor activity in multiple other tumor types, nivolumab was the first anti-PD-1 agent to improve OS in recurrent/metastatic (R/M) HNSCC progressing after a first-line platinum-based therapy in the Checkmate 141 trial with a 32% reduction in the risk of death (205, 232). OS was 7.7 months compared with 5.1 months with chemotherapy. Benefit was greater in ≥1% PD-L1 positive (PD-L1+) TPS (tumor cell membrane positivity for PD-L1 or tumor proportion score) patients with an OS of 8.2 versus 4.7 months. Pembrolizumab is the other agent to show efficacy in the second line in the Keynote 0-40 trial (233). Median OS was 8.4 vs 6.9 months with a hazard ratio (HR) of 0.80. Contrary to the Checkmate 141 essay, crossover was allowed. Subgroup analysis shows that, for PD-L1 TPS ≥ 50% patients, survival was significantly increased from 7.9 to 11.6 months with immunotherapy, whereas there was no difference for the PD-L1<50% population.

This same agent is the new standard of front-line therapy in R/M HNSCC following the results of the Keynote-048 trial comparing pembrolizumab alone or in combination with platinum-based and 5FU chemotherapy to the EXTREME standard of care protocol (cisplatin or carboplatin, 5-fluorouracile (5FU) and cetuximab) (234). Survival was significantly increased with pembrolizumab compared with the EXTREME regimen for PD-L1 ≥20 CPS (expression on both tumor cells and immune cells in the microenvironment or combined positive score) patients (14.7 vs. 11 months) and PD-L1 ≥1 CPS patients (12.3 vs. 10.4 months) but not in the total population (11.5 vs. 10.7 months). It is important to note that the experimental treatment was deleterious for some patients in the beginning with more deaths occurring in the first 6 months. Pembrolizumab added to chemotherapy significantly improved OS in the total population with safety comparable to the EXTREME arm (13 vs. 10.7 months). The median duration of response was impressive with pembrolizumab at 22.6 months (vs. 4.5 for EXTREME). Tolerance was also far better with immunotherapy. The FDA approved pembrolizumab for use in combination with platinum and fluorouracil for all patients and as a single agent for patients with a CPS ≥1. In Europe, the EMA approved the use of pembrolizumab alone or in combination for patients with a CPS ≥1. Unlike PD-L1, blockade of cytotoxic T-lymphocyte antigen 4 (CTLA4) as monotherapy has not proven beneficial in HNSCC.

Evidently, monotherapy with ICI seems to be a losing battle despite providing substantial clinical improvements over the previous standards of care. The majority of patients do not respond to treatment, and durable responses are observed only in a minority (generally less than one third) of patients.

### Resistance to Immune Checkpoint Inhibitors

Resistance to ICI can be primary (never-responder patients) or secondary (acquired after a certain amount of time of response). It can also be classified as intrinsic to tumor cells (cancer cells directly induce immune resistance *via* various mechanisms) or extrinsic (other cells or factors mediate immune resistance).

HNSCC hijacks numerous cellular and molecular immunomodulatory pathways to evade recognition and eradication by the immune system. Mechanisms of immune evasion include direct T cell suppression with surface or soluble inhibitory factors, decreased immune stimulation, and the recruitment of immuno-suppressive cell populations (231). In this section, we review the different types of resistance reported in HNSCC and present some of the currently studied strategies to overcome them.

### Intrinsic Resistance

#### Tumor Immunogenicity and Antigen Presentation

HNSCC is one of the cancers with the highest levels of tumor mutational burden (TMB), accompanying elevated neoantigen expression (220). These tumoral neoantigens that derive from nonsynonymous mutations drive (T lymphocytes) TL cytotoxic response against tumor cells. In that sense, a positive correlation between response and TMB was found in a recent meta-analysis (235). Constant interactions between immune and cancer cells can result in a selection of subclones lacking the expression of neoantigens, subsequently resulting in poor immunogenicity and decreasing efficacy of ICI (236). This could explain how some HNSCC tumors with high TMB are unresponsive to ICI.

Furthermore, deficiencies in antigen presentation can result in primary or acquired resistance to ICI as shown in multiple studies (237, 238). This includes downregulation of MHC class I (MHC-I) and loss of function (e.g., truncating mutations) of β2-microglobulin (238). HNSCC has been shown to alter neoantigen presentation and processing by altering key genes, such as signal transducer and activator of signal (STAT) 1 and other antigen processing machinery components (239, 240).

Combining ICI with radiation therapy is a promising strategy as radiotherapy leads to an increased rate of neoantigens and antigen presentation induced by DC activation, increased cytokine production, and tumor cell death, promoting a TIL phenotype (241, 242). Chemotherapy increases antigen release upon cell death and, thus, the priming of cytotoxic TL (243), and this was the rational in combining platinum-based chemotherapy with ICI in the Keynote-048 trial that resulted in added benefit (234).

Emerging novel therapies include oncolytic virus therapy and cancer vaccines with tumor peptides or DCs (244, 245). Their aim is to enhance antigen presentation and TL priming. Oncolytic viruses can also directly infect and induce lysis of tumor cells. Talimogene laherparepvec (TVEC), which is derived from herpes simplex virus type 1, is currently under evaluation in combination with pembrolizumab in the Keynote-137 trial in R/M HNSCC patients. Other novel therapeutics, such as toll-like receptor (TLR) agonists (NCT02521870) and adoptive cell therapy (NCT03247309), are also being evaluated in this same context.

#### Oncogenic Pathways

Aberrations in canonical oncogenic pathways can change the TME by altering cytokine production and immune cell composition. These include the MAPK (246), WNT/β-catenin (247), and PI3K pathways (248). The activation of the latter creates an immunosuppressive TME. Combined inhibition of PD-1 and PI3K in a preclinical model of HNSCC demonstrates a synergistic growth inhibitory effect and increased survival of mice by activating an immunostimulatory transcriptional program, enhancing T cell cytotoxicity and expression of proinflammatory cytokines (249).

#### Soluble Molecules

HNSCC cells can also avoid T cell rejection by secreting immunosuppressive exosomes containing transforming growth factor (TGF) β, PD-1, and CTLA4, which impair T and NK cell functions and upregulate Tregs (250). They can also produce and secrete protumoral cytokines, including TGF-β, interleukin (IL)-6, and IL-10 (251). Tumor cells can overexpress Indoleamine 2,3-dioxygenase 1 (IDO1), a rate-limiting enzyme that converts tryptophan to kynurenine, leading to an immune suppression through T cell apoptosis and loss of function. In a study of the immune microenvironment of HPV-negative OSCC from never-smoker and never-drinker (NSND) patients, it was suggested that blockade of IDO1 and PD-1/PD-L1 could insure a higher clinical benefit. However, a phase-III clinical trial evaluating Epacadostat, an IDO inhibitor, in combination with Pembrolizumab was halted after a similar trial in melanoma revealed no improvement compared with the control arm (252).

### Extrinsic Resistance

#### Inhibitory Checkpoint Molecules

Overexpression of alternative immune checkpoints can be a source of adaptive resistance to ICI. These receptors serve to limit effector functions of the immune system to prevent autoimmunity in a normal state. Multiple inhibitory immune checkpoint receptors with different cell distributions and expression patterns have been described, including PD-L1, CTLA4, lymphocyte-activation gene 3 (LAG-3), T-cell immunoglobulin, mucin domain-3 protein (TIM-3), B and T lymphocyte attenuator (BTLA), V-domain immunoglobulin-containing suppressor of T cell activation (VISTA), and T cell immunoreceptor tyrosine-based inhibition motif domain (TIGIT) (253, 254).

PD-L1 status has been shown to be partially correlated with response to ICI in HNSCC, but complete responses have been observed in PD-L1-negative patients (232–234). CTLA4 is upregulated in HNSCC tumor cells and enriched on Treg TILs (255). These cells are a subset of CD4+ T cells with immunosuppressive effects through various humoral and cellular mechanisms, such as CTLA4-mediated suppression of antigen-presenting cells (256). LAG-3 is expressed on activated CD4+ and CD8+ T cells, NK cells, B cells, and DCs (257). It binds with major histocompatibility complex class II (MHCII) and is highly expressed on Tregs. It was shown that blockade of LAG-3 decreases the inhibitory function of these cells (257). TIM-3 is expressed on both T and NK cells and binds with galectin-9 (258). When specifically coexpressed with PD-1, TIM-3 is the signature of an exhausted T cell phenotype (259).

Because these alternate coinhibitory receptors induce T-cell exhaustion (231), they have been identified as a putative strategy to overcome resistance to PD-1 in previous and many ongoing studies. Based on these observations, two essays of ICI anti-PD1 and anti-CTLA4 combination in HNSCC have been reported to date (260). The CONDOR trial compared outcomes of patients who had low/negative PD-L1 (TPS<25%) tumors and had progressed after first-line platinum-containing therapy. Patients were treated with either durvalumab, an anti-PDL1 Ab, or tremelimumab, an anti-CTLA4 Ab, or the combination (260). Results were deceiving as there was no significant increase in response rate (RR) (7.8% vs. 9.2%), PFS (2 vs. 1.9 months) or OS (7.6 vs. 6 months) compared with durvalumab alone. One toxic death from acute respiratory failure was attributed to the combination regimen. The same combination failed to improve survival regardless of PD-L1 status in the EAGLE trial (261). Monotherapy with the anti-CTLA4 agent in CONDOR appeared clearly inefficient with a 1.6% RR, a 1.9 and 5.5 median PFS and OS, respectively (260). The authors hypothesize that the lack of efficacy of tremelimumab may be in part related to its mechanism of action, which, as an IgG2 Ab, does not cause lysis of regulatory T cells through ADCC, contrary to what is observed with ipilimumab, another anti-CTLA4 agent (262).

Inhibitors of other checkpoint molecules, such as TIM-3 and LAG-3, are still in earlier phases of development. For example, blockade of TIM-3, whose expression is synonymous to T cell exhaustion, is efficient in producing an antitumoral T cell response in a mouse model of HNSCC (263).

Inhibitory checkpoints can also be expressed on the surface of innate immune cells, such as NK cells. The inhibitory killer immunoglobulin-like receptor (KIR) 2DL-1, -2, -3 receptors, which partially control NK cell activation upon binding with their ligands, primarily human leukocyte antigen-C (HLA-C) molecules, can be targeted by Lirilumab, a fully human IgG4 monoclonal Ab. PD-1 blocking on T cells can induce the release of cytokines, such as IL-2, that enhance NK cell function, whereas blockade of KIR can result in the secretion of IFN-γ that may boost T cell–mediated antitumor responses (264). This rationale of NK–T cell crosstalk led to the testing of the combination in phase-1/2 trials (265).

#### Stimulatory Agonist Molecules

The balance between coinhibitory and costimulatory signals is what determines the state of the immune response. Costimulatory agonists include Ox40, 4-1BB, inducible T cell co-stimulator (ICOS), and CD40. Ox40 is expressed on the surface of T cells and promotes proliferation and IFN-y production. It is shown to be present in HNSCC, but expression of its ligand (Ox40L) is reduced, rendering this pathway ineffective (266). ICOS is expressed on the same cells, promoting a Th2 response [62], and 4-1BB, present on the surface of activated T cells, NK cells, and DCs, is shown to be downregulated in HNSCC patients (267).

Many promising ongoing trials are evaluating receptor agonists in order to reverse resistance to ICI and augment durable responses. For example, agonists of ICOS are being evaluated in combination with anti-PD-1, anti-CTLA-4, and chemotherapy in various tumors, including HNSCC (NCT03693612, NCT02904226). In addition, urelumab (an agonistic 4-1BB monoclonal antibody), is evaluated in combination with cetuximab (NCT02110082) in R/M HNSCC patients.

#### Immunosuppressive Cells

In addition to all the complex interactions between coinhibitory and costimulatory pathways, immunosuppressive cells can modulate the immune response and create a protumoral environment *via* multiple diverse mechanisms (268). Their recruitment to the TME is regulated by HNSCC cells. MDSCs, Tregs, and TAMs all modulate NK and T cell responses to various degrees.

MDSCs are an immature myeloid cell population that promotes HNSCC invasiveness, angiogenesis, and metastasis (269, 270) by secreting immunosuppressive enzymes, such as enzymes arginase 1 (Arg-1) and nitric oxide synthase (iNOS) (251). Their presence correlates with poor outcomes with ICI as shown in melanoma patients (271). Monoclonal Ab and small molecule inhibitors that inhibit MSDC functions are currently investigated in R/M HNSCC patients in various clinical trials.

Tregs facilitate self-tolerance through direct contact and inhibitory cytokines, such as IL-10 and TGF-β (272), they also play a key role in immune evasion in HNSCC. These cells preferentially express CCR4 (believed to have a major role in the recruitment of Tregs to the TME), which is being targeted with an anti-CCR4 monoclonal antibody, mogamulizumab, in different tumor types, including HNSCC.

TAMs, particularly M2 macrophages, enhance tumor angiogenesis, motility, growth, and immune evasion by secreting protumoral cytokines in the TME [69]. Their presence in HNSCC is associated with poor prognosis (273). Antibodies and small molecules that inhibit colony-stimulatory factor 1 receptor (CSF1R) binding with CSF1, which serves recruitment to the tumor of M2 TAMs, are currently underway in various advanced solid tumors, including HNSCC (NCT02526017).

## Conclusions and Perspectives

Treatment of the majority of patients with HNSCC requires multimodality approaches. Currently, cetuximab is used in the clinical routine as a radiation sensitizer alone or in combination with chemotherapy for the treatment of patients with recurrent or metastatic disease. More recently, pembrolizumab was approved as a first-line therapy in patients who present with metastatic disease, and treatment with either pembrolizumab or nivolumab is used in the setting of cisplatin-refractory recurrent or metastatic HNSCC. Despite the encouraging results observed in some patients, tumor responses observed in most patients are only partial and are systematically followed by acquired resistance due the reactivation of oncogenic signaling, leading to tumor regrowth, as discussed in this review.

Most of the developments toward understanding HNSCC have fallen short of clinically meaningful discoveries, highlighting an urgent need for more effective therapies to improve treatment outcomes. The increasing knowledge on the genomic driver alterations in HNSCC enables their use as predictive markers of targeted therapy regimens, currently evaluated in clinical trials, which are shown to improve survival and tumor response in subgroups of patients (274, 275). For instance, late-phase clinical trials show that *HRAS*-mutant HNSCC patients (8% of HNSCC) treated with tipifarnib, a selective farnesyltransferase inhibitor, shows promising outcomes with an overall response rate (ORR) of 42.9% with a median duration of response of 14.7 months (275). The Akt/mTOR axis is activated in most HNSCC, particularly in surrounding normal mucosa, and is associated with recurrences. In this context, a phase-II trial (NCT01111058) shows significant improvement in 1-year PFS in patients with locally advanced HNSCC treated with everolimus (276). More recently, Xevinapant, an investigational inhibitor of apoptosis protein (IAP) blocker, showed prolonged OS when added to standard chemoradiotherapy for locally advanced head and neck squamous cell carcinoma. Based on these results, Xevinapant received breakthrough therapy status from the FDA for the first-line treatment of HNSCC in September 2020.

The molecular heterogeneity of HNSCC has hampered the identification of specific targets and, thus, the development of targeted therapies for this group of tumors (51). Indeed, much of the difficulty in studying and treating HNSCC lies in the fact that they are a heterogeneous group of cancers arising from distinct anatomic subsites that display distinct molecular features and are associated with diverse risk factors. However, these diseases are treated uniformly and with limited success.

Genome-wide expression profiling led to the identification of four robust molecular classes of HNSCC (277–279). In this classification, the “classical,” “basal,” and “mesenchymal” subtypes exhibit canonical genomic alterations, such as nuclear factor erythroid 2−related factor 2 (NFE2L2) mutations and high expression of genes in oxidative stress response pathways, high frequency of *HRAS* mutations, and upregulation of EMT-related genes, respectively (278, 279). Of note, multiple findings have led to increased interest in the mechanisms by which cancer cells undergoing EMT or oscillating within the EMT spectrum might contribute to immune escape through various routes. The “atypical” subtype contains a high proportion of HPV+ tumors, who themselves are very heterogenous and can be subclassified into HPV-KRT (HPV-keratinocyte differentiation and oxidative reduction process) or HPV-IMU (HPV-immune response and mesenchymal cell differentiation) tumors (69). These different subtypes of HNSCC may harbor different patterns of sensitivity to oncogenic-driven targeted therapy and radiotherapy (280, 281); however, the clinical implication of these subtypes is currently unknown.

More recently, based on the gene expression profiles of 1368 patients with SCC in the Cancer Genome Atlas (TCGA), Li B et al. (282) proposed six immune subtypes, including an immune-cold subtype, an immune-hot subtype, a subtype dominated by M2-polarized macrophages, and three other immune subtypes with more diverse immunologic features. Their association with response or resistance to immunotherapy is unclear.

Complementary strategies to assess the molecular programs that are specific to each histological subtype or anatomical location of HNSCC may benefit from comprehensive analyses of patient samples (283) to identify molecular vulnerabilities and, thus, enable rapid clinical deployment to guide therapeutic decisions. Furthermore, single-cell transcriptomics may help revealing intratumoral heterogeneity (ITH) (284, 285) with subtypes as well as identifying cell populations that drive drug resistance. Spatial transcriptomics might also be an informative approach to enable simultaneous capture of the distribution and localization of the different components of the TME and, thus, better understand its interaction in response to treatment. Finally, the establishment of relevant preclinical models of HNSCC (ref) that reflect the disease at the genetic, histological, and functional level may provide a tool to study the molecular modifiers of response to therapies currently used in the clinical routine or tested in clinical trials.

Overall, understanding the molecular vulnerabilities of HNSCC may contribute to identify and therapeutically target residual disease and prevent or delay the evolution of acquired resistance. Of note, acknowledging that drug resistance depends not only upon cancer cells but also upon the TME might enable the identification of potential drug targets to limit cancer cell adaptation to therapy.

## Author Contributions

SO-C, JB, AK, JF, and PS: contributed to this paper with the design. SO-C, JB, AK: literature search. SO-C, JB, AK, JF, and PS: drafting. SO-C, JB, AK, JF, and PS: revision and editing. SO-C, JB, AK, JF, and PS: final approval. All authors contributed to the article and approved the submitted version.

## Funding

This work was supported by funding from the Integrated Cancer Research Site LYriCAN (INCa-DGOS-Inserm_12563). SO-C received funding from the Fondation ARC pour la Recherche sur le Cancer (ARCPJA22020060002212). JB received funding from Nuovo Soldati Foundation (2019) and ITMO Cancer 2020, Formation à la Recherche Fondamentale et Translationnelle en Cancérologie.

## Conflict of Interest

PS receives research grants from HTG Molecular Diagnostics, Inivata, Bristol-Myers Squibb, Roche Molecular Diagnostics, Roche, AstraZeneca, Novartis, Bristol-Myers Squibb Foundation, and Illumina.

The remaining authors declare that the research was conducted in the absence of any commercial or financial relationships that could be construed as a potential conflict of interest.

## Abbreviations

EGFR, Epidermal Growth Factor Receptor; PTPRS, The protein tyrosine phosphatase receptor S (interacts with EGFR and inactivates EGFR); CTX, cetuximab; TGF-α, transforming growth factor alpha; TGF-ß, transforming growth factor beta; EGF, Epidermal Growth Factor; HB-EGF, heparin-binding growth factor; JAK2, Janus kinase 2; MAPK, Mitogen-activated protein kinases; MET, Mesenchymal Epithelial Transition; HER2 or ERbB2, human epidermal growth factor receptor 2; HER3 or ERbB3, human epidermal growth factor receptor 3; ROR2, receptor tyrosine kinase-like orphan receptor 2; IGF-1R, Insulin growth factor type 1 receptor; VEGFR, vascular endothelial growth factor receptor; PI3K, Phosphoinositide 3-kinases; mTOR, Mechanistic Target Of Rapamycin Kinase; SMAD4, Mothers against decapentaplegic homolog 4; DNA, deoxyribonucleic acid; RNA, ribonucleic acid; EMT, Epithelial-to-mesenchymal transition; T cells, T lymphocyte; T regs, regulatory T lymphocyte.
